# Different Measures of Structural Similarity Tap Different Aspects of Visual Object Processing

**DOI:** 10.3389/fpsyg.2017.01404

**Published:** 2017-08-17

**Authors:** Christian Gerlach

**Affiliations:** Department of Psychology, University of Southern Denmark Odense Odense, Denmark

**Keywords:** category-effects, classification, structural similarity, superordinate categorization, visual complexity, visual object recognition

## Abstract

The structural similarity of objects has been an important variable in explaining why some objects are easier to categorize at a superordinate level than to individuate, and also why some patients with brain injury have more difficulties in recognizing natural (structurally similar) objects than artifacts (structurally distinct objects). In spite of its merits as an explanatory variable, structural similarity is not a unitary construct, and it has been operationalized in different ways. Furthermore, even though measures of structural similarity have been successful in explaining task and category-effects, this has been based more on implication than on direct empirical demonstrations. Here, the direct influence of two different measures of structural similarity, *contour overlap* and *within-item structural diversity*, on object individuation (object decision) and superordinate categorization performance is examined. Both measures can account for performance differences across objects, but in different conditions. It is argued that this reflects differences between the measures in whether they tap: (i) global or local shape characteristics, and (ii) between- or within-category structural similarity.

## Introduction

Structural similarity can loosely be defined as the shape overlap between two or more objects; the greater the overlap, the more similar they are. From this it follows that the more structurally similar objects are, the *harder* it should be to tell them apart; a process termed individuation. This indeed appears to be the case. Gerlach et al. (2015) for example showed that the more structurally similar two objects are, the more difficult it is to decide whether they are the same or different objects. This behavioral effect was further shown to be tightly tied to activation in occipitotemporal brain regions subserving visual object processing; the greater the structural similarity, the higher the activation. Perhaps less intuitively, it also turns out that the more structurally similar objects are, the *easier* it is to decide whether they belong to the same superordinate class; an effect which probably arises because objects that belong to the same category are typically more similar in shape than objects that belong to different categories (Gerlach et al., 2015). Hence, structural similarity can act as a proxy for category membership (Rosch, 1999) and is considered important for inductive inference (Sloutsky, 2009; Godwin and Fisher, 2015). These opposing effects of (structural) similarity on object individuation and object categorization have also been expressed in mathematical terms as two different decision rules in the Generalized Context Model (Nosofsky, 1986, 1987), which is an exemplar model of classification. Furthermore, it has been suggested that differences in structural similarity can also account for category-specific recognition disorders following brain damage (Gerlach, 2009), and for category-effects in normal visual object processing; that is, for observations that natural objects (animals, vegetables, etc.) are processed *less* efficiently than artifacts (vehicles, tools, etc.) when they must be individuated, as in object naming, but *more* efficiently than artifacts when assigned to a superordinate category, as in superordinate categorization (Riddoch and Humphreys, 1987; Price and Humphreys, 1989; Gerlach et al., 2000; Kiefer, 2001; Gale et al., 2006; Gerlach and Gainotti, 2016). In both cases, it has been hypothesized that the category-effects reflect that natural objects are more structurally similar than artifacts (Humphreys et al., 1988; Gerlach, 2016); a suggestion clearly compatible with the notion that structural similarity is harmful for object individuation but beneficial for superordinate categorization.

In spite of its merits as an explanatory variable, structural similarity is not a unitary construct, and it has been operationalized quite differently by different authors. Humphreys et al. (1988), who were the first to consider the role of structural similarity in category-specific disorders, for example offered two measures of structural similarity: Contour overlap (CO), and number of common attributes. The measure of CO was generated by: (i) Normalizing all the pictures of objects in the Snodgrass and Vanderwart (1980) corpus for orientation and size, (ii) overlaying each picture on a grid with pictures of every other object from the same category (the categories being animals, birds, body parts, buildings, clothes, crustacea, fruit, furniture, implements, insects, vegetables, and vehicles), and (iii) calculating the average overlap between the pictures on their bounding contour –that is, on their outline shape without consideration of internal details– as a function of the amount of contour in each target picture. Ratings of common attributes were based on participants who were asked to list, from the category name, the attributes in common across exemplars within the 12 different categories listed above. Natural objects were found to score higher than artifacts on both measures, suggesting that natural objects are generally more structurally similar than artifacts. Similar results have been found by Tranel et al. (1997) with a related measure of within-category shape overlap based on silhouettes –which also captures similarity in global shape– and by Cree and McRae (2003) based on feature production norms from a corpus of 541 concepts. In contrast to these studies, Laws and Gale (2002) (see also Laws et al., 2003) found that artifacts were actually more structurally similar than natural objects when internal details of stimuli were also measured. This finding was based on Euclidean Overlap; a measure of pixel overlap across all pairs of pictures in the Snodgrass and Vanderwart (1980) corpus.

It is clear that the measures described above cover quite a spectrum. The Euclidean Overlap measure used by Laws and Gale (2002) –which was based on raw pixel overlap– is more low-level that the size and orientation normalized CO measure derived by Humphreys et al. (1988). In comparison to both of these measures, common attribute ratings are rather high-level being based on features abstracted from memory rather than physical images. Clearly, these measures are likely to tap different aspects of structural similarity (Humphreys and Riddoch, 2002).

A better understanding of what aspects of structural similarity these different measures may capture can be gleaned from a study by Op de Beeck et al. (2008). They used a stimulus set comprising nine artificial shape categories which could be grouped based on either of two orthogonal similarity dimensions. One dimension was rather low-level being a pixel-based measure of overlap not unlike the one derived by Tranel et al. (1997), and thus capturing the overlap in global shape across stimuli; the dimension also captured by the CO measure. The other dimension captured similarity in terms of features (which could be characterized as either spiked, smoothed, or cubed). Grouping the categories along the latter dimension corresponded rather closely to a multidimensional-scaling-derived solution based on pair-wise similarity ratings provided by the participants, suggesting that perceived similarity was based on abstraction of the objects’ features rather than on physical overlap among the objects’ global shapes. Furthermore, fMRI revealed that there were no reliable patterns of “preference” for low- or high-level based similarity in visual brain regions V1, V2, V3, or V4v, although activation in these areas tended to be better associated with low-level than with high-level measures of similarity. In comparison, the activation pattern in the lateral occipital complex (LOC) was clearly related to ratings of perceived (feature-based) shape similarity (Op de Beeck et al., 2008). A similar finding was reported by Weber et al. (2009). They found that the pairwise similarity of neural responses in LOC to pictures of mammals correlated highly with subjective pairwise similarity ratings of the same set of mammals but not with measures of pixel-based similarity. Together these findings suggest a posterior-to-anterior anatomical axis gradient in similarity sensitivity with posterior regions being sensitive to low-level based similarity, reflecting physical global shape overlap, and more anterior areas being sensitive to high-level based similarity, reflecting more abstract feature-based similarity. This posterior-to-anterior axis similarity gradient has also been demonstrated with use of photographs of real objects (Bracci and Op de Beeck, 2016).

It has recently been suggested that the difference between globally- and feature-based structural similarity, which here is tied to low- and high-level measures, respectively, may offer an explanation for the observation that pixel- and contour-based measures of similarity have been successful in predicting category-effects in classification at the superordinate level (e.g., that natural objects in general are assigned faster to a superordinate category than artifacts), but not category-effects in classification at basic and subordinate levels where objects must be individuated (e.g., that natural objects are named less efficiently than artifacts) (Gerlach, 2016). The explanation rests on the assumption that low-level measures of structural similarity, based on pixel and contour overlap, capture similarity at a coarse scale (global shape) which is sufficient to support superordinate classifications, but which is simply too crude to serve for subordinate or even basic level classifications, which in turn may rely on more fine-grained differentiation among features. It has further been proposed that if this assumption is correct, low-level similarity measures, reflecting global shape overlap, should correlate with superordinate classification performance, whereas higher-level (feature-based) similarity should correlate with performance in tasks requiring object individuation such as basic level (dog vs. cat) or subordinate (Fox Terrier vs. Rottweiler) naming (Gerlach, 2016).

The purpose of the present study is to test these predictions directly by examining how structural similarity affects the processing of individual objects at two levels of classification. This is done by examining the performance of 457 participants in two different tasks: (i) *difficult object decision* (deciding whether pictures represent real objects or non-objects), which requires object individuation at a finer scale than basic level naming (Gerlach and Toft, 2011), and (ii) *superordinate categorization* (deciding whether stimuli represent natural objects or artifacts). The real objects in the object decision task were the same objects which were presented in the superordinate categorization task allowing for a direct comparison of items across tasks. As a relatively low-level measure of (physical) structural similarity the CO measure developed by Humphreys et al. (1988), which was described above, was used. As a high-level measure of structural similarity the *within-item structural diversity* (WSD) measure developed by Turnbull and Laws (2000) was used. Turnbull and Laws (2000) presented participants with the name of an object (e.g., dog, fork) and asked them to rate the extent to which instances with that name had similar structural representations (on a scale of 1–5; 1 = very dissimilar; 5 = very similar). Hence, elephants score higher on this measure than chairs as chairs come in a variety of shapes whereas elephants do not. The WSD measure can be considered a high-level measure of structural similarity because it is based on memories of objects rather than on the physical properties of objects (drawings) such as the CO measure. Both measures (CO and WSD) were based on items in the Snodgrass and Vanderwart (1980) corpus, which also served as stimuli in the object decision and superordinate categorization tasks examined here.

The specific hypotheses tested, and their premises, were: (1) If CO reflects global (physical) structural similarity among objects from different basic level categories (e.g., different types of animals), and if global structural similarity is beneficial for making superordinate categorizations, a negative correlation between CO and reaction time (RT) in the superordinate categorization task should be found; the greater the CO, the faster the categorization. (2) If WSD reflects more abstract feature-based structural similarity among objects belonging to the same basic level categories (e.g., different dogs), and if structural similarity is harmful for object individuation, a positive correlation between WSD and RT in the object decision task should be found; the greater the structural similarity, the longer it will take to individuate the object.

If the predicted effects are found, it will suggest that structural similarity, or at least different aspects of structural similarity, exerts opposing effects on individuation and superordinate categorization.

We have recently demonstrated that another variable; visual complexity, acts in a similar way in that high visual complexity is beneficial for superordinate categorization but harmful for individuation (Gerlach and Marques, 2014). In this study, visual complexity was indexed by the total number of concavities on the bounding contour of each picture combined with a measure of the pictures’ internal details. The reason why visual complexity exerts opposing effects on superordinate categorization and object individuation is further addressed in the discussion below. Here it suffices to say that visual complexity and visual similarity are likely to reflect different dimensions of objects (Panis and Wagemans, 2009). As an example, fruits and mammals are both categories with many structurally similar members but mammals are more visually complex than fruits, which again are more structurally similar but less visually complex than members of the category furniture. It is, however, not known how visual complexity and structural similarity exert their influence. Do the effects modulate each other or are they additive? Hence, as a last objective the relative influences of structural similarity and visual complexity on superordinate categorization and object individuation were examined.

## Materials and Methods

### Participants

The data comprised responses from a total of 457 individuals (mean age 23, range 18–57 years, 331 females). Following data trimming (see below) the dataset comprised responses from a total of 409 individuals (mean age 23, range 18–57 years, 298 females).

The dataset has been collected over a 5 year period (2011–2015), and parts of the dataset have formed basis for two published papers. As mentioned above, Gerlach and Marques (2014) examined the role of visual complexity on object individuation and superordinate categorization (*N* = 184), whereas Gerlach and Gainotti (2016) examined whether there were any gender differences in category-effects which they did not find support for (*N* = 366). The participants were students who performed the experiments as part of their education; a course in cognitive psychology. The course is approved by the study board at the Department of Psychology, University of Southern Denmark, and the experiments conducted do not require formal ethical approval/registration according to Danish Law and the institutional requirements. Prior to participation the students were informed that data collected in the experiments might be used in an anonymous form in future publications. Participants were free to opt-out if they wished, and participation in the experiments was taken as consent.

### Design

Each participant first performed the categorization task followed by the object decision task. In the categorization task the participants were to press the M-key if the stimulus depicted an artifact and the N-key if it depicted a natural object. In the object decision task the participants were instructed to press the M-key if the stimulus represented a real object and the N-key if it represented a non-object. The participants were asked to respond as fast and as accurately as possible. Prior to data collection the participants performed a practice version of the upcoming task. Stimuli used for practice were not used in the actual experimental conditions.

### Stimuli

In the categorization task 80 pictures were presented. These pictures were selected from the set by Snodgrass and Vanderwart (1980) and comprised 40 artifacts and 40 natural objects. The two sets of objects were matched with respect to image agreement, visual complexity, and familiarity based on the norms collected by Snodgrass and Vanderwart (1980). However, the two sets of objects differed significantly in both CO [*t*(59.98) = -6.2, *p* < 0.001] and WSD [*t*(78) = -3.4, *p* < 0.001], with natural objects scoring higher –being more similar– than artifacts on both measures. The CO measure was available for 35 of the natural objects and for 33 of the artifacts. The WSD measure was available for all 80 items.

**FIGURE 1:**
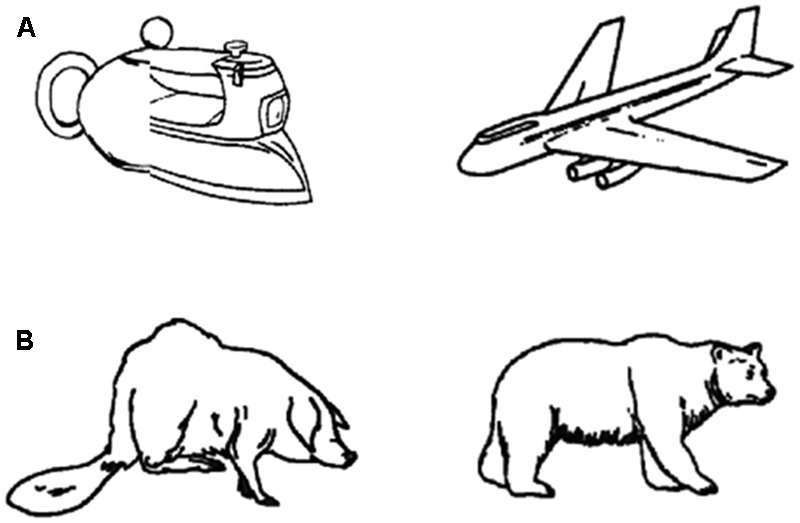
Examples of stimuli used in the object decision task. **(A)** Chimeric non-objects, and **(B)** real objects. The real objects were also used in the superordinate categorization task.

In the object decision task 160 stimuli were presented: 80 chimeric non-objects and 80 real objects. The real objects were the same as used in the categorization task. The 80 chimeric drawings of non-objects used in the difficult object decision tasks were selected mainly from the set made by Lloyd-Jones and Humphreys (1997). These stimuli are line-drawings of closed figures made by exchanging parts belonging to objects from the same category (**Figure 1**). The non-objects were composed by parts from objects that were not used as real objects. The order of stimuli was randomized in each task.

### Procedure

All stimuli subtended 3–5°of visual angle and were presented centrally on a white background on a PC-monitor. The stimuli were displayed until response. The interval between response and presentation of the next stimulus was 1 s. RTs were recorded via keyboard.

### Statistical Analysis

The RT data were positively skewed and contained several outliers. This was addressed using the same procedure as Gerlach and Marques (2014). First the data was trimmed by excluding any participant who had a RT which fell above/below 2 SDs from the mean of the whole sample for any of the four conditions (object decision with artifacts, object decision with natural objects, categorization of artifacts, and categorization of natural objects). Data from 48 participants were removed [10.5% of the data, which is within the recommended limits; Ratcliff (1993)]. Next the same procedure was performed across items. This resulted in the removal of 7 items [8.8% of the data, which again is within the recommended limits; Ratcliff (1993)]: 2 items from object decision with natural objects [#159, 201], and 2 items from object decision with artifacts [#189, 213], 2 items from the categorization of natural objects [#241, 252], and 1 item from the categorization of artifacts [#58]. The items removed were exactly the same as the ones removed in the study by Gerlach and Marques (2014). This is not just a consequence of the fact that part of the current dataset (40%) comprised the data used by Gerlach and Marques (2014). When trimming was performed on the data not used in the study by Gerlach and Marques (2014) (*n* = 273), all of the seven pictures besides #241 deviated more than 2 SDs from the sample means. The trimmed data (over both participants and items) were subsequently log transformed (LogRT). Following these procedures the data did not depart significantly from normality (Kolmogorov–Smirnov, all *p*’s > 0.05).

The first series of analyses examined effects of task and category on accuracy scores across the four conditions, and also included a two-way ANOVA on the LogRTs with the factors Task (categorization vs. object decision) and Category (artifacts vs. natural objects). These analyses are performed over both participants and items. Analyses by participants identify effects that are reliable across participants, but which could in principle be driven by a few items which are not representative for the whole set of items. In contrast, analyses by items identify effects that are reliable across items, but which could potentially be driven by a few participants who are not representative for the whole group of participants. Hence, an effect of category, which is found significant across both participants (denoted *F*_1_) and items (denoted *F*_2_), will signify an effect which is representative for the majority of the participants tested and for the majority of the examined items from the category. While the analysis of Category is not of direct importance here, it is included because the dataset gives a unique possibility to test category-effects across the two tasks in a very large sample (*N* = 409).

Given that the only purpose of the non-objects in the present study was to ensure detailed shape processing of the real objects, the analyses of LogRTs presented below are all based on correct responses to *real* objects only (Gerlach and Toft, 2011). Likewise, accuracy scores are also based on responses to *real* objects only.

In the second series of analyses it is first examined whether the two measures of structural similarity –CO and WSD– correlate with each other. Then it is examined whether they correlate with performance on the object decision and the categorization tasks. The measure of CO is available for 63 of the 73 items that survived the trimming procedure. The measure of WSD is available for all. To make comparisons of the two measures comparable the analyses are confined to the 63 items for which both measures are available. However, the correlations for the WSD measure on all 73 items are also reported. The correlations reported are based on the Pearson product-moment correlation coefficient, and their 95% confidence intervals are computed by means of bias corrected and accelerated bootstrap analyses with 1000 samples.

In the final series of analyses, the relative influence of structural similarity and visual complexity on object individuation and categorization is examined by means of multiple regression. The measure of visual complexity used here is based purely on objective image statistics: The total number of concavities on the bounding contour of each picture combined with a measure of its internal details [the ratio (internal pixels)/(total pixels)]. For a more detailed discussion of this measure compared with other measures of visual complexity the reader is referred to Gerlach and Marques (2014).

## Results

### Effects of Task and Category

#### Accuracy

Analyses by participants (Wilcoxon signed-rank test) revealed a significant difference in accuracy between artifacts and natural objects in both the object decision task (*Z* = -4.67, *r* = 0.23, *p* < 0.001) and the superordinate categorization task (*Z* = -4.95, *r* = 0.25, *p* = 0.001); with accuracy being higher for artifacts in both conditions. Accuracy was also significantly higher in the categorization task than in the object decision task for both artifacts (*Z* = -8.95, *r* = 0.44, *p* < 0.001) and natural objects (*Z* = -8.43, *r* = 0.42, *p* < 0.001).

Analyses by items revealed a significant difference in accuracy between artifacts and natural objects in the object decision task (Mann–Whitney test, *U* = 229.5, *r* = 0.57, *p* < 0.001), and in the categorization task (Mann–Whitney test, *U* = 429.5, *r* = 0.32, *p* = 0.001). Also, for natural objects, accuracy was significantly higher in the categorization task than in the object decision task (Wilcoxon signed-rank test, exact test, *Z* = -3.99, *r* = 0.47, *p* < 0.001). For artifacts this difference was not significant (Wilcoxon signed-rank test, exact test, *Z* = -1.1, *r* = 0.13, *p* = 0.28).

#### Log RTs

The ANOVA analyses revealed a significant main effect of Task [*F*_1_(1,408) = 334.95, *MS*_e_ = 1067, $\eta_{p}^{2}$ = 0.45, *p* < 0.001; *F*_2_(1,71) = 95.32, *MS*_e_ = 0.08, $\eta_{p}^{2}$ = 0.57, *p* < 0.001], with longer logRTs during object decision compared with categorization, an effect of Category that was significant over participants [*F*_1_(1,408) = 10.12, *MS*_e_ = 0.007, $\eta_{p}^{2}$ = 0.02, *p* < 0.001] but not over items [*F*_2_(1,71) = 0.03, *MS*_e_ = 0.00, $\eta_{p}^{2}$ = 0.0, *p* = 0.87], and a significant interaction between Task and Category [*F*_1_(1,408) = 316.1, *MS*_e_ = 0.234, $\eta_{p}^{2}$ = 0.44, *p* < 0.001; *F*_2_(1,71) = 32.93, *MS*_e_ = 0.027, $\eta_{p}^{2}$ = 0.32, *p* < 0.001], with the difference between tasks being greater for natural objects than for artifacts. Pairwise comparisons over both items and participants (*t*-tests) revealed that all simple main effects were significant (all *p*’s < 0.03). See **Table 1** for details concerning RTs, LogRTs, and accuracy.

**Table 1:** Number of observations (n), median accuracy in %, mean RTs and mean log-transformed RTs (LogRT) in the object decision task and the categorization task computed across items and participants.

	*n*	Accuracy in %	RT	LogRT
**Object decision (Real objects) over items**				
Artifacts	37	98 (89–100)	765 (80)	2.88 (0.04)
Natural objects	36	96 (91–98)	800 (66)	2.90 (0.04)
**Categorization over items**				
Artifacts	37	98 (94–100)	729 (31)	2.86 (0.02)
Natural objects	36	98 (94–99)	684 (36)	2.83 (0.02)
**Object decision (Real objects) over participants**				
Artifacts	409	97.5 (80–100)	762 (108)	2.88 (0.06)
Natural objects	409	95 (67.5–100)	799 (119)	2.90 (0.06)
**Categorization over participants**				
Artifacts	409	100 (72.5–100)	718 (111)	2.85 (0.07)
Natural objects	409	97.5 (75–100)	672 (102)	2.82 (0.07)

*Range for accuracy in % and standard deviations for RTs and LogRTs are given in brackets. The differences between natural objects and artifacts in accuracy and latency were significant in both tasks (object decision and categorization) and across both items and participants (all *p*’s < 0.01).*

The finding of faster LogRT for natural objects than for artifacts in the superordinate categorization task in the context of lower accuracy for natural objects than for artifacts could in principle reflect a speed-accuracy trade-off. To address this formally, it was examined whether the difference in LogRT between categories in the superordinate categorization task could be accounted for by differences in accuracy. This was done by computing the correlation between LogRT and category while controlling for accuracy. Performed over participants, the correlation between LogRT and category was unchanged after controlling for accuracy (*r* = -0.21 in both cases). For the same analysis over items, the correlation between LogRT and category increased from *r* = -0.47 to *r* = -0.69 after controlling for accuracy. Hence, there is nothing to suggest that the faster LogRTs for natural objects compared with artifacts in the superordinate task reflects that the participants have reduced their RT to natural objects relative to artifacts at the cost of reducing their accuracy with natural objects. Note also that the significant decrease in latency for natural objects in the superordinate categorization task compared with the object decision task is mirrored by a similar reduction in error rate; just as the case was for artifacts (over participants).

Considered together the results from the analyses examining effects of category are rather clear: Natural objects are processed less efficiently than artifacts when the demand on perceptual differentiation is high (object decision), but this disadvantage is reversed (latency)/reduced (accuracy) when the demand on perceptual differentiation is lowered (superordinate categorization).

### Effects of Structural Similarity on Object Individuation and Superordinate Categorization

There was a reliable positive correlation between the CO and the WSD measures for the items examined here {*r*(63) = 0.32, 95% CI [11, 49], *p* < 0.05}. In comparison, the correlation between the CO and the WSD for the whole sample of Snodgrass and Vanderwart items for which both measures are available is *r*(219) = 0.24 (95% CI [13, 35], *p* < 0.001). This suggests that the association between the two measures is not only found for the subset of items examined here.

**FIGURE 2:**
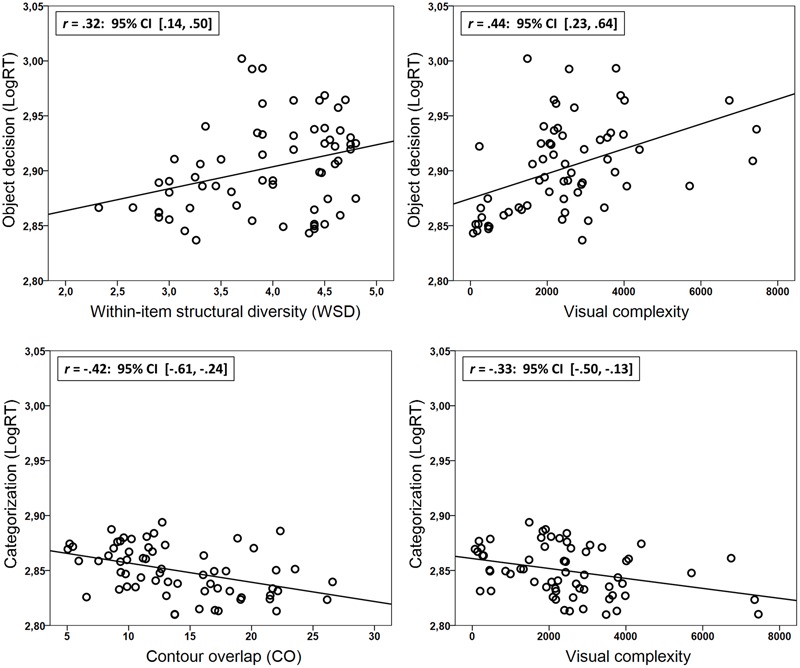
Scatterplots showing the correlations (zero-order) between structural similarity (WSD and CO)/visual complexity and LogRT in the object decision task **(Upper)** and the categorization task **(Lower)**. Also shown are the regression lines, the Pearson correlation coefficients (*r*) and their associated 95% CI’s computed by means of bias corrected and accelerated bootstrap analyses with 1000 samples.

Contour overlap did not correlate reliably with LogRT in the object decision task {*r*(63) = 0.02, 95% CI [-18, 23], *p* = 0.86} whereas WSD did {*r*(63) = 0.32, 95% CI [14, 50], *p* < 0.05} [For the full set of items surviving the trimming procedure (*N* = 73), the correlation between object decision LogRT and the WSD measure was *r* = 0.32, 95% CI [14, 50], *p* < 0.01]. For superordinate categorization the reverse was found in that CO correlated reliably with LogRT {*r*(63) = -0.42, 95% CI [-61, -24], *p* < 0.001} whereas WSD did not {*r*(63) = -0.1, 95% CI [-31, 13], *p* = 0.45}{For the full set of items surviving the trimming procedure (*N* = 73), the correlation between superordinate categorization LogRT and the WSD measure was *r* = -0.03, 95% CI [-26, 19], *p* = 0.78}. See **Figure 2** upper and lower left panels for scatterplots showing the relationship between LogRT and structural similarity (WSD and CO) in the object decision task and the superordinate categorization task.

As described above natural objects and artifacts differed in terms CO and WSD, with natural objects scoring higher than artifacts on both measures. Hence, the category-effects observed in the first analysis could in principle be driven by differences between the two categories in CO and WSD. To examine this possibility, two new ANCOVA analyses were performed on all items for which CO and WSD measures were available (33 natural objects and 30 artifacts), with the two measures used as covariates. Prior to entering WSD as a covariate in the analysis of the object decision task, there was a significant effect of category (*F*_2_ = 5.66, *MS*_e_ = 0.009, $\eta_{p}^{2}$ = 0.09, *p* < 0.05). However, following adjustment for the effect of WSD, the effect of category was no longer significant (*F*_2_ = 1.84, *MS*_e_ = 0.003, $\eta_{p}^{2}$ = 0.03, *p* = 0.18). For superordinate categorization, the effect of category was significant both before (*F*_2_ = 33.84, *MS*_e_ = 0.011, $\eta_{p}^{2}$ = 0.36, *p* < 0.001) and after adjusting for CO (*F*_2_ = 17.05, *MS*_e_ = 0.006, $\eta_{p}^{2}$ = 0.22, *p* < 0.001).

In summary, increasing levels of structural similarity affected object processing in a negative manner during individuation (WSD), but in a positive manner during superordinate categorization (CO), and these effects of structural similarity were able to account for much of the variance otherwise attributed to category (natural objects vs. artifacts).

### The Relative Influence of Structural Similarity and Visual Complexity on Object Individuation and Superordinate Categorization

Given that WSD correlated reliably with performance in the object decision task, and given that visual complexity has been shown to affect performance in this task (Gerlach and Marques, 2014), the relative influence of these measures on performance was examined by means of a hierarchal multiple regression analysis with two steps. Step one included only WSD as a predictor of object individuation performance, whereas step two also included visual complexity as a predictor. As can be seen from **Table 2**, both WSD and visual complexity were reliable predictors of object individuation performance, and hence addition of visual complexity increased the fit of the regression model from *R*^2^ = 0.1 to *R*^2^ = 0.28.

**Table 2:** Linear model of predictors of object individuation performance (object decision).

	*b*	95% CI	*SE b*	β
**Step 1**				
Constant	2.824	2.786, 2.868	0.0212	
Within-Item structural similarity	0.020	0.008, 0.03	0.005	0.32
**Step 2**				
Constant	2.805	2.766, 2.846	0.0212	
Within-Item structural similarity	0.018	0.007, 0.028	0.005	0.29
Visual complexity	0.000011	0.000006, 0.000018	0.000003	0.42

*95% confidence intervals and standard errors are estimated by means of bias corrected and accelerated bootstrap analyses with 1000 samples. *R*^*2*^ = 0.10 for Step 1; Δ*R*^*2*^ = 0.28 for Step 2 (*p*’s < 0.05).*

Given that CO correlated reliably with performance in the superordinate categorization task, and given that visual complexity has been also shown to affect performance in this task (Gerlach and Marques, 2014), the relative influence of these measures on performance was also examined by means of a hierarchal multiple regression analysis with two steps. Step one included only CO as a predictor of categorization performance, whereas step two also included visual complexity as a predictor. As can be seen from **Table 3**, both CO and visual complexity were reliable predictors of superordinate categorization performance, and hence addition of visual complexity increased the fit of the regression model from *R*^2^ = 0.18 to *R^2^* = 0.28.

**Table 3:** Linear model of predictors of superordinate categorization performance.

	*b*	95% CI	*SE b*	β
**Step 1**				
Constant	2.874	2.862, 2.887	0.0065	
Contour overlap	-0.002	-0.003, -0.001	0.0004	-0.42
**Step 2**				
Constant	2.885	2.870, 2.903	0.0072	
Contour overlap	-0.002	-0.003, -0.001	0.0004	-0.42
Visual complexity	-0.000004	-0.000007, -0.000002	0.000001	-0.33

*95% confidence intervals and standard errors are estimated by means of bias corrected and accelerated bootstrap analyses with 1000 samples. *R*^*2*^ = 0.18 for Step 1; Δ*R*^*2*^ = 0.28 for Step 2 (*p*’s < 0.01).*

It is worth noting that adding visual complexity as a predictor to the two regression models did not affect the strength of the correlations found between object decision performance and the WSD measure on the one hand and superordinate categorization performance and the CO measure on the other. In other words, the effect of structural similarity on object decision and superordinate categorization performances is not influenced by differences in visual complexity suggesting that effects of structural similarity and visual complexity are additive.

## Discussion

The object decision task was more difficult to perform than the superordinate categorization task. This is compatible with the assumption that it requires less object individuation to classify an object at a superordinate level than it does to decide whether it represents a real object or a chimeric non-object (Gerlach et al., 2000). As in previous studies, it is also found that task type (object decision vs. categorization) interacts with category (natural objects vs. artifacts), with natural objects being processed faster than artifacts in the superordinate categorization task but more slowly in the object decision task (Gerlach, 2009).

A lot of evidence suggest that this interaction between task type and category can be explained by assuming that natural objects are more structurally similar than artifacts, and that high structural similarity is advantageous when objects must be assigned to a superordinate category but disadvantageous when objects must be individuated (Gerlach, 2009). However, as discussed in the Introduction, evidence directly supporting the role of structural similarity in visual object processing has been inconsistent. It has recently been proposed that some of this inconsistency may reflect that low-level measures of structural similarity, based on pixel and CO, may capture similarity at a coarse level (global shape) that is informative for making categorizations at the superordinate level. In comparison, the type of similarity that affects classification at the basic and subordinate level is likely to be captured by more abstract measures, such as number of common parts and distinctive features, which may not correspond in any direct way to statistics derived from physical images (Gerlach, 2016). This proposal was tested directly here by examining how two different measures of structural similarity correlated with performance in a superordinate categorization task, which requires little object individuation, and an object decision task which requires comparably more object individuation. The first similarity measure examined is a relatively low-level measure based on the amount of CO between objects belonging to the same superordinate class (e.g., animals) (Humphreys et al., 1988). The other high-level measure examined, termed WSD (Turnbull and Laws, 2000), reflects how structurally similar people judge different instances of the same basic level category, i.e., DOG, to be.

As predicted, the CO measure correlated significantly with performance in the superordinate categorization task accounting for 18% of the variance; the higher the CO, the faster the classification. In comparison, CO had no reliable effect on object individuation performance (object decision). For the WSD measure the reverse was found. WSD had no reliable effect on superordinate categorization performance but it accounted for 10% of the variance in object individuation performance, with low structural similarity being associated with faster performance. These findings directly support the suggestion that structural similarity exerts opposing effects on superordinate categorization and object individuation. Moreover, analyses of covariance demonstrated that category (natural object vs. artifact) did not explain any variance in the object decision task that could not be accounted for by differences in WSD, and that adjusting for CO caused the amount of variance explained by category in the superordinate categorization task to drop considerably. Both observations support the notion that category-effects may, at least partly, be accounted for by difference in structural similarity between natural objects and artifacts. Finally, the present results reflect performance differences with individual objects, rather than performance differences based on mean similarity ratings for given categories of objects (e.g., natural objects vs. artifacts).

In spite of the different effects of CO and WSD on task performance, there was a moderate positive correlation between the measures (*r* = 0.32). This relationship may reflect that basic level categories, whose members are rather similar according to the WSD measure, such as DOG, also tend to resemble other basic level categories in structure, such as TIGER, with which they form superordinate categories, such as ANIMAL. It is the latter aspect which is most clearly captured by the CO measure.

It has recently been described how another measure –visual complexity– seems to work in the same manner as structural similarity (Gerlach and Marques, 2014). Using the same tasks as described here (and a subset of the same participants; *n* = 184), Gerlach and Marques (2014) showed that high levels of visual complexity were beneficial for fast superordinate categorization performance (*r* = -0.31) but harmful for fast object individuation (*r* = 0.43). These similarities between visual complexity and structural similarity prompted an examination of the conjoint effects of these variables on superordinate categorization and object individuation in the present sample. This was done by means of multiple regression with WSD and visual complexity as predictors of object individuation performance (object decision), and with CO and visual complexity as predictors of superordinate categorization performance.

The results of these analyses showed that WSD and visual complexity had similar, but independent, effects on object individuation performance (object decision), and that adding visual complexity as a predictor increased the amount of explained variance from 10 to 28%. For both predictors, higher scores on these variables were associated with poorer performance. Likewise, CO and visual complexity had similar, but independent, effects on superordinate categorization performance, and adding visual complexity as a predictor increased the amount of explained variance from 18 to 28%. For both predictors, higher scores on these variables were associated with better performance. Hence, structural similarity and visual complexity cause similar and opposing effects on superordinate categorization and object individuation, and the effects of these variables are additive.

Both effects of structural similarity and visual complexity can be accounted for in the framework of the PACE model which was originally advanced in order to account for category-effects in visual object processing (Gerlach, 2009). To see how, this model will be described briefly. According to PACE, visual object classification is based on two operations: *Shape configuration* and *selection*. The first is the binding of visual elements into elaborate shape descriptions which specifies the relationships between the parts. This shape description can then be matched with structural representations stored in visual long-term memory (VLTM). The matching process is conceived of as a race between VLTM representations competing for selection. The VLTM representation that matches the configured shape description the best wins the race, i.e., is selected. The match criterion is considered task dependent. Hence, when fine-grained discrimination is required, as in classification at a subordinate level, there will be an elaborate match criterion. In a task like superordinate categorization on the other hand, which requires gross perceptual processing only, a more lax match criterion will be sufficient. When the shape description is successfully matched with a VLTM representation based on a given criterion, the object is classified as a particular sort of instance. The more specific the match criterion, the more loops of VLTM access and shape configuration must be undertaken to gain sufficient evidence for correct classification. Thus, the degree of perceptual differentiation—or object individuation—required by a given task depends directly on the nature of the match criterion.

At the stage of selection, then, objects with high structural similarity are thus disadvantaged compared with structurally distinct objects when the demand on perceptual differentiation is high. This is so because structurally similar objects activate a greater number of related VLTM representations that compete for selection than do structurally distinct objects. However, at the stage of shape configuration, objects characterized by high structural similarity may enjoy an advantage. This assumption is based on the following premises: (i) Global shape characteristics may be processed before local shape characteristics, providing an initial frame in which local details are later embedded; (ii) global shape characteristics are more diagnostic of basic level object identity for structurally similar than for structurally distinct objects; and (iii) information concerning object identity can be used to augment shape configuration in a top-down manner [for evidence supporting these assumptions see Gerlach (2016)]. Hence, global shape characteristics may be more supportive for the shape configuration of structurally similar than for the shape configuration of structurally distinct objects.

A final –and central– assumption in the PACE model is that shape configuration does not precede access to VLTM. Rather, there is a first pass access to VLTM representations based on the global outline of the stimulus. This first pass will yield initial hypotheses concerning the likely identity of the stimulus which can then be used in a top-down manner to support the build-up of a more detailed description of the stimulus; a description which can serve as input for a more specific match with VLTM representations (Gerlach and Toft, 2011).

In PACE then, structural similarity is thus assumed to be harmful for object individuation. If, on the other hand, the demand on perceptual differentiation is low –that is, when objects need not be individuated– PACE predicts better performance with structurally similar objects. This is so because: (i) The global shape of structurally similar objects is more diagnostic of category membership than the global shapes of structurally distinct objects, (ii) the shapes of structurally similar objects are more easily configured than the shapes of structurally distinct objects, while (iii) there is no need for selection based on fine-grained discriminations. Hence, PACE can readily account for the opposing effects of structural similarity on object decision and superordinate categorization found here.

As demonstrated by Gerlach and Marques (2014), PACE can also account for the effect of visual complexity. Specifically they argued that high visual complexity will be: (i) Beneficial for selection because objects of high complexity, due to their richness of information, activate fewer candidate representations in VLTM that need to be differentiated than less complex objects, but (ii) harmful for shape configuration because it is more difficult to form (elaborate) perceptual representations of complex objects than of less complex objects [for similar arguments see Panis and Wagemans (2009) and Torfs et al. (2010)]. Hence, during superordinate categorization, high visual complexity is advantageous because there is little need for object individuation. In comparison, during object decision there is such a need, causing high visual complexity to be harmful.

Finally, PACE also offers a possible explanation for a somewhat puzzling aspect of the current results. As argued above, it is not surprising that the CO measure did not correlate with object individuation, as it taps structural similarity *among* rather than *within* basic-level categories. What is more surprising is that the WSD measure did not correlate with both object individuation performance *and* superordinate categorization performance. After all, if members of a given basic-level category are highly similar, and if high structural similarity is beneficial for superordinate categorization, why then are objects which are highly structurally similar according to the WSD measure not assigned to a superordinate category faster than objects which are more structurally distinct? To answer this question, consider first that the CO measure primarily reflects similarity in global shape characteristics, being based only on the objects’ bounding contour and not on internal details. In comparison, the WSD measure is likely to reflect similarity in terms of features (local shape characteristics), cf. the finding by Op de Beeck et al. (2008) that people spontaneously, that is without specific instructions other than to rate visual similarity, weigh features more than global shape. If this is so, and if superordinate classification can be accomplished based primarily on global shape characteristics (Collin and McMullen, 2005), this may explain why WSD does not correlate with superordinate classification performance even though it taps structural similarity and affects object individuation. In PACE, this idea can be tied to the assumption that global shape, and hence global shape similarity, dominates early on in object processing, driving the first pass access to VLTM representations. If object classification can be accomplished based on this first pass, within-category similarity may not matter much. Accordingly, within-category similarity will only affect the shape configuration process, and subsequent recurrent processing, if the demand on perceptual differentiation increases, and then in a proportion contingent on the degree of perceptual differentiation required.

The present findings bear some resemblance to visual search studies which have also shown that form-based target-distractor similarity modulates search performance (Duncan and Humphreys, 1989; Tollner et al., 2015); the higher the similarity the less efficient the search becomes. This modulation may reflect similarity in both local shape characteristics (details/features) and global shape characteristics (the configuration of local shape characteristics). Interestingly, when the target is defined by a configuration of local shapes which forms a pregnant global shape (e.g., a Kanizsa figure), search is less affected by target-distractor similarity than when the local configuration does not form a pregnant shape (Conci et al., 2007). In other words, target-distractor similarity cannot simply be predicted by summing the similarity between the individual local elements but also rests on higher order shape characteristics carried by the configuration of the local elements. This is not unlike the role that the PACE model ascribes to global shape in mediating the first pass access to VLTM representations.

## Conclusion

The present study finds direct support for the assumption that structural similarity is beneficial for superordinate categorization but harmful for object individuation. In comparison with previous studies, the present results reflect differences in performance with individual and common objects, rather than performance differences based on mean similarity ratings for given categories of objects (e.g., natural objects vs. artifacts) or artificial stimuli. It is also suggested that different measures of structural similarity may capture different aspects of this construct. In particular, the two measures examined here: CO and WSD, may differentially tap variations along two dimensions: (i) global (CO) versus local shape characteristics (WSD), and (ii) between-category (CO) versus within-category structural similarity (WSD). Both dimensions are likely to be relevant at different stages in visual object processing, which is also reflected in the explanations offered for the present findings. A firmer understanding of their relative contributions, however, must await future studies that can tease these aspects apart.

## Author Contributions

The author confirms being the sole contributor of this work and approved it for publication.

## Conflict of Interest Statement

The author declares that the research was conducted in the absence of any commercial or financial relationships that could be construed as a potential conflict of interest.
